# How Sample Size Impacts Probabilistic Stimulation Maps in Deep Brain Stimulation

**DOI:** 10.3390/brainsci13050756

**Published:** 2023-05-03

**Authors:** Teresa Nordin, Patric Blomstedt, Simone Hemm, Karin Wårdell

**Affiliations:** 1 Department of Biomedical Engineering, Linköping University, 58185 Linköping, Sweden; 2 Department of Clinical Science, Neuroscience, Umeå University, 90185 Umeå, Sweden; 3 Institute for Medical Engineering and Medical Informatics, School of Life Sciences, University of Applied Sciences and Arts Northwestern Switzerland, 4132 Muttenz, Switzerland

**Keywords:** deep brain stimulation (DBS), probabilistic stimulation maps (PSM), finite element method (FEM), electric field simulation

## Abstract

Probabilistic stimulation maps of deep brain stimulation (DBS) effect based on voxel-wise statistics (*p*-maps) have increased in literature over the last decade. These *p*-maps require correction for Type-1 errors due to multiple testing based on the same data. Some analyses do not reach overall significance, and this study aims to evaluate the impact of sample size on *p*-map computation. A dataset of 61 essential tremor patients treated with DBS was used for the investigation. Each patient contributed with four stimulation settings, one for each contact. From the dataset, 5 to 61 patients were randomly sampled with replacement for computation of *p*-maps and extraction of high- and low-improvement volumes. For each sample size, the process was iterated 20 times with new samples generating in total 1140 maps. The overall *p*-value corrected for multiple comparisons, significance volumes, and dice coefficients (DC) of the volumes within each sample size were evaluated. With less than 30 patients (120 simulations) in the sample, the variation in overall significance was larger and the median significance volumes increased with sample size. Above 120 simulations, the trends stabilize but present some variations in cluster location, with a highest median DC of 0.73 for *n* = 57. The variation in location was mainly related to the region between the high- and low-improvement clusters. In conclusion, *p*-maps created with small sample sizes should be evaluated with caution, and above 120 simulations in single-center studies are probably required for stable results.

## 1. Introduction

Deep brain stimulation (DBS) provides electrical pulses to alleviate symptoms of different movement disorders, psychiatric disorders, pain conditions, etc. Depending on the disorder and pathophysiology, different target structures have been suggested, some more established than others. However, the disorders are often complex and multifactorial, and the optimal location of stimulation within the different targets is still debated [1].

Probabilistic stimulation maps (PSM) were introduced to objectively investigate the optimal stimulation location. PSMs combine an estimate of the volume of tissue activated (VTA) by the stimulation and the clinical outcome to create a statistical mapping of the DBS effect for individual patients [2], or patient groups [3,4,5]. A common procedure for VTA estimation is electric field simulation based on the location of the DBS lead in the anatomy and the stimulation settings [6].

When PSMs are created by applying statistical tests in each voxel, a multitude of comparisons are conducted on the same data. The resulting probability map (*p*-map) will therefore require correction for multiple comparisons (Type 1 error). There are several methods for this, e.g., permutation procedures [7,8,9] or false-discovery rate (FDR) [10,11] where permutation test is more common. Some analyses fail to survive the correction for multiple comparisons, and the limited number of patients (e.g., approximately 10–30 patients) in several DBS-PSM studies can be questioned [12,13,14].

A recent publication reviewing data from several studies on essential tremor (ET) also showed a notable variation in the optimal stimulation spot [13]. A future possibility is to predict the effect in new patients based on PSMs, but that requires reliable results of the mappings. Several publications have included predictive algorithms based on PSMs [11,13,15,16], but they explain the interpatient variability poorly. The influence of the amount of data on the mapping results has not yet been explored.

This paper aims to investigate the variation in *p*-maps from DBS with regard to sample size. The evaluated parameters are the overall *p*-map significance together with the volume and location of significant voxels.

## 2. Materials and Methods

Thorough descriptions of the clinical procedure [17] and PSM method [3] have been published previously. Section 2.1, Section 2.2, Section 2.3 and Section 2.4 will contain a brief summary.

### 2.1. Clinical Data

Data from 61 patients with ET operated on in the posterior subthalamic area (caudal zona incerta) between 2003–2016 (lead 3387 or 3389, Medtronic, Minneapolis, MN, USA) were retrieved. The leads in the left hemisphere only were used for analysis. Written informed consent was obtained from the patients and the study was approved by the local Ethics Committee at Umeå University (Dnr. 01-123M, 2017/122-31). The patient data included pre-operative MRI (T1- and T2-weighted), postoperative CT for lead localization, stimulation parameters, and clinical improvement according to the essential tremor rating scale (ETRS) [18]. The analysis was based on data from a monopolar review 1 year after surgery, i.e., each contact of the DBS quadripolar lead was tested for clinical improvement using standardized pulse width (60 μs) and repetition frequency (145 Hz). During this test, the amplitude was increased in increments of 0.2–0.3 V up to 5 V, and the lowest amplitude generating the best outcome according to ETRS items 5/6 and 11–14 was noted. This gave a total of 244 stimulations.

### 2.2. Electric Field Simulations

To estimate the tissue impacted by the stimulation, the electric field was simulated with the finite element method (FEM) (COMSOL Multiphysics v. 5.5, COMSOL AB, Stockholm, Sweden) [19]. This method uses the volume conductor model with a heterogeneous isotropic tissue conductivity based on segmentation of the pre-operative T_2_-weighted MRI. The DBS lead was modeled according to manufacturer specification and placed in the tissue volume according to the post-operative CT. A 250 µm thick peri-electrode space surrounding the lead was modeled as white brain matter [20]. The simulations were computed in a box of size 100 × 100 × 100 mm with a physics-controlled mesh (the finest element size closest to the lead).

### 2.3. Image Normalization

For group analysis, all patients were transformed to the same space, a group-specific template [3]. As a first step, the preoperative MRI was aligned to the MNI nLin-aSym 2009b [21] using an affine transform. Subsequently, 8 iterations of non-linear registration and template update were performed to refine the template. The resulting template is an average representation of the patient group. The electric field norm from the simulations was then transformed to the template space using the warp fields computed during the normalization. As a last step, the electric fields were sampled on the template voxel grid to facilitate spatial group analysis.

### 2.4. Probabilistic Stimulation Maps

The resampled electric fields were used to compute probabilistic maps (*p*-maps) with a voxel-wise statistical test [3]. Before analysis, the electric field was truncated at 0.2 V/mm since lower magnitudes are assumed to not invoke any clinical response. A t-statistics based on the electric field magnitude and the clinical improvement in tremor reduction (TR) was computed in each voxel according to

(1) $$t_{i}=\frac{{\hat{TR}}_{i}-70}{\sqrt{\frac{s_{i}^{2}}{N_{i}}}}$$

where *N* is the number of simulations above 0.2 V/mm in voxel *i*, *s* is the standard deviation, and $\hat{TR}$ (%) is the weighted mean tremor reduction. Voxels with *N_i_* less than 10% of all the sampled simulations or 5, whichever was greatest, were excluded from the analysis, i.e., the threshold was dependent on the sample size. To evaluate the effect of the exclusion threshold, complete analyses were also run with fixed thresholds of *N_i_* ≤ 5 and *N_i_* ≤ 12 corresponding to the minimum threshold and the 10% threshold for 30 patients. The $\hat{TR}$, weighted with the electric field norm ***E*** (V/m) over the stimulation amplitude *A* (V), was calculated in each voxel *i* according to

(2) $${\hat{TR}}_{i}=\frac{\sum_{k}\frac{E_{ik}}{A_{k}}\cdot {TR}_{k}}{\sum_{k}\frac{E_{ik}}{A_{k}}}$$

where *k* denotes the stimulation. A voxel *p*-value < 0.05 was regarded as significantly different from a 70% improvement, which corresponds to the mean improvement of the stimulations in the investigated population. Voxels with positive t-statistics were grouped into a high-improvement cluster, and voxels with negative t-statistics were grouped into a low-improvement cluster.

To correct for type 1 error of multiple comparisons, a permutation test was applied where the improvement values were permuted 200 times, first by permuting all leads and then by permuting the simulations within each lead. The permuted distributions were used to recompute *p*-maps and compared with the true distribution using an overall statistic. Significant *p*-value (*p* < 0.05) indicates an overall significance of the true *p*-map. The same test with the 10% exclusion threshold was repeated with 1000 permutations to separate the effect of sample size and the number of permutations.

### 2.5. Evaluation of Sample Size

To evaluate the effect of sample size, the *p*-maps were computed by sampling 5–61 (*n*) patients, i.e., 20–244 simulations, using random sampling with replacement. The sampling was done on a patient level since there might be a dependency within stimulations from the same patient and to have an equal frequency of the stimulating contacts. For each sample size, 20 different samplings were made resulting in 1140 *p*-maps.

### 2.6. Data Analysis

The resulting *p*-maps were evaluated by investigating the overall *p*-value from the permutation test in the different sample sizes. Furthermore, cluster volumes (V) from the *p*-maps were extracted representing the high-improvement voxels (voxels > 70% improvement) and low-improvement voxels (voxels < 70% improvement). For reference, the total analyzed volume and mean number of simulations per voxel were computed. The dice coefficient (DC) (Equation (3)) was used for evaluating the spatial variation between the 20 clusters resulting from the same sample size, for high and low improvement separately.

(3) $$DC=2\frac{\left|V_{X}⋂V_{Y}\right|}{\left|V_{X}\right|+\left|V_{Y}\right|}\;X=1,\ldots ,19;\;Y=X+1,\ldots ,20$$

The result for each sample size is presented as the median and interquartile range (IQR). For evaluations over all the sample sizes, the results are presented as mean ± standard deviation.

## 3. Results

**Figure 1:**
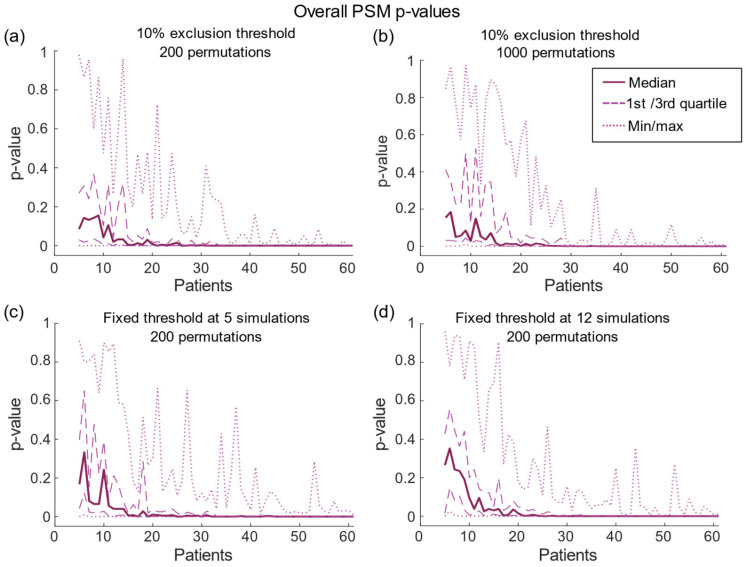
The overall *p*-value for the computed *p*-maps with (**a**) 200 and (**b**) 1000 permutations with 10% exclusion threshold and with 200 permutations for fixed exclusion thresholds of (**c**) 5 simulations and (**d**) 12 simulations. All data are shown with the median (straight line) and the variation is displayed with the quartiles (dashed line) and max/min (dotted line).

The result for the overall *p*-values can be seen in Figure 1. With 200 permutations, the variation in overall *p*-value was higher for small sample sizes (max IQR = 0.333, *n* = 8) but reached low *p*-values around 20–30 patients, i.e., 80 to 120 simulations, with only a few outliers (IQR = 0, *n* > 35). A similar result was seen with 1000 permutations (Figure 1b) (max IQR = 0.495, *n* = 11; IQR = 0, *n* > 35) and for the fixed exclusion thresholds (Figure 1c,d) (max IQR = 0.530/0.413, *n* = 6/5; IQR = 0, *n* > 35/35 for threshold of 5 and 12, respectively) but with slightly larger variation for low sample sizes.

**Figure 2:**
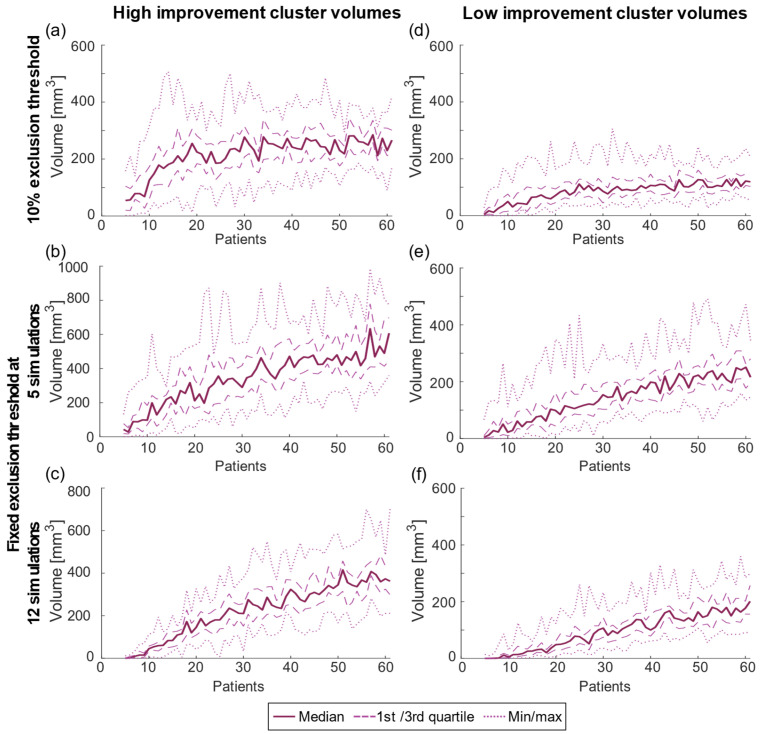
Computed cluster volumes for (**a**–**c**) high improvement and (**d**–**f**) low improvement for the 10% exclusion threshold (top row), and fixed threshold at 5 simulations (second row) and 12 simulations (bottom row).

**Figure 3:**
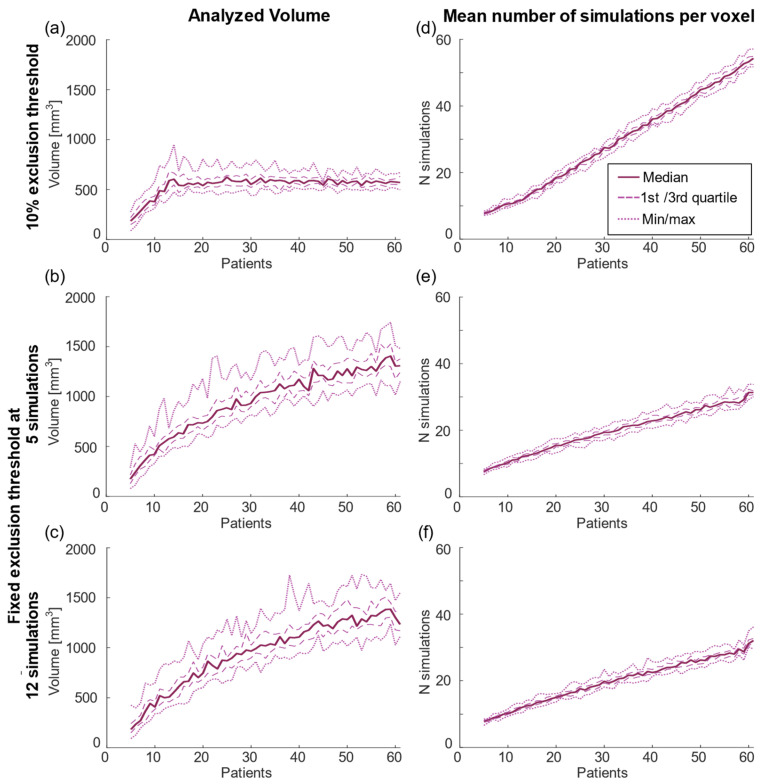
The total volume included in the statistical analysis (**a**–**c**), and the mean number of simulations in the analyzed voxels (Ni) (**d**–**f**) for the 10% exclusion threshold (top row), and fixed threshold at 5 simulations (second row) and 12 simulations (bottom row).

The variation of the cluster volumes for both high and low improvement was similar over the different sample sizes, but a low exclusion threshold generates increasing variability (Table 1) (Figure 2). The absolute volumes increased with sample size and reached stabilization around *n* = 30 for both the high- and low-improvement clusters but this trend was less pronounced for the fixed exclusion threshold analyses (Figure 2b,c,e,f). For reference, Figure 3 shows the total volume included in the statistical analysis and its mean occurrence of simulations. While the mean number of simulations per voxel increased in a nearly linear way, the analyzed volume was stable after reaching the point where the threshold increases with sample size, i.e., at 12 patients. When using a fixed threshold analysis, the analyzed volume increased through the entire analysis, but more pronounced for low sample sizes.

**Figure 4:**
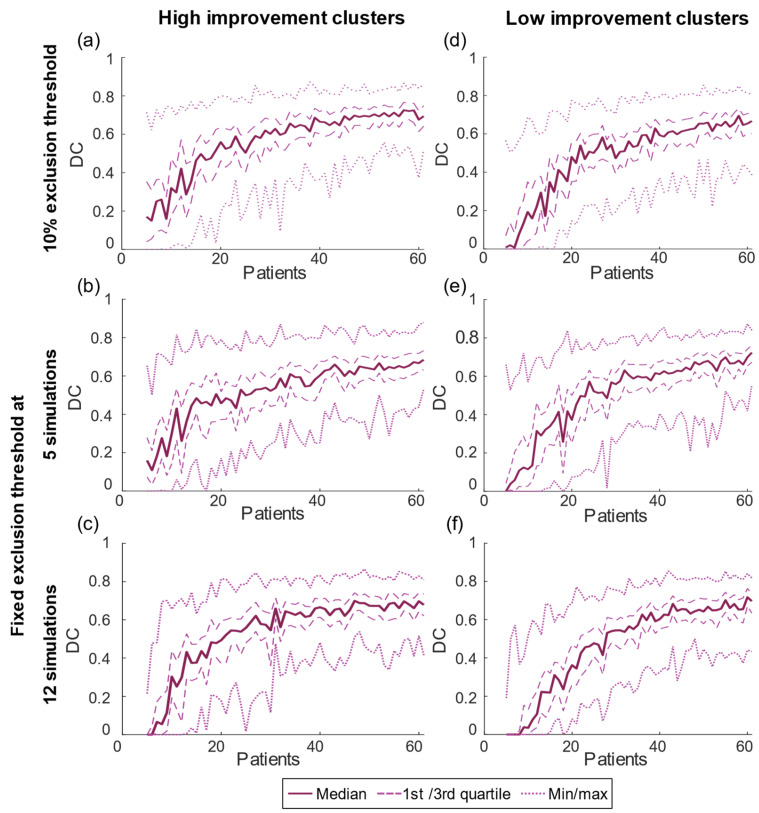
Dice coefficient for the clusters within each sample size for the (**a**–**c**) high-improvement clusters and (**d**–**f**) low-improvement clusters with the 10% exclusion threshold (top row), and fixed threshold at 5 simulations (second row) and 12 simulations (bottom row).

The DC increased over the entire span of sample sizes but more rapidly in the beginning (*n* < 40) (Figure 4). The median values of the DC reached a maximum of 0.68–0.72 for the different analyses, and the variation was similar for all sample sizes (Table 2). Similar trends and effect sizes were seen for all analyses independent of the threshold selection.

**Figure 5:**
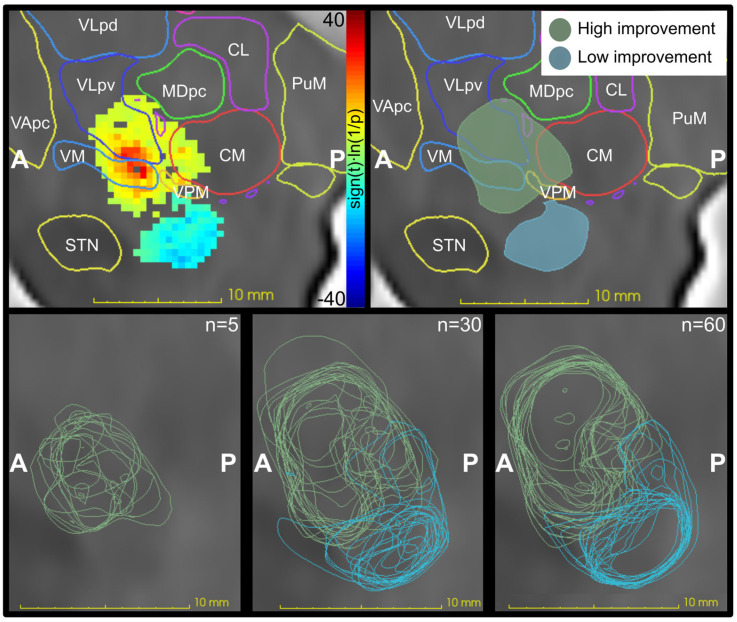
The top row shows on the left an example (*n* = 60) of a *p*-map and on the right the same *p*-map as high- and low-improvement clusters in sagittal views together with outlines from a digital version of Morel’s atlas [22,23]. The bottom row shows sagittal views of the outlines from all high- and low-improvement clusters computed with 5, 30, and 60 patients. The clusters converge with increasing sample size and the location is expanded in the superior direction. All images are from the analysis with the 10% exclusion threshold. CL = central lateral nucleus, CM = centromedian nucleus, MDpc = mediodorsal nucleus (parvocellular division), PuM = medial pulvinar, STN = subthalamic nucleus, VApc = ventral anterior nucleus (parvocellular division), VLpd = ventral lateral posterior nucleus (dorsal division), VLpv = ventral lateral posterior nucleus (ventral division), VM = ventral medial nucleus, VPM = ventral posterior medial nucleus, A = anterior, P = posterior, n = number of included patients.

Figure 5 shows an example of a *p*-map and visualizes the localization of all clusters for sampling 5, 30, and 60 patients for the 10% threshold analysis. The clusters were in the vicinity of each other with a clear trend that the cluster centers converge to one region with increasing samples. The discrepancy in the location shown by the DC was mainly due to variations in the outer regions in the inferior-posterior direction for high-improvement volumes and the superior-anterior direction for low-improvement volumes.

## 4. Discussion

The present study has investigated the influence of the number of included DBS patients in *p*-map computations. A total of 244 simulations from 61 ET patients were included based on stimulation information obtained during a monopolar review 1 year after surgery.

This study shows that a small sample size generates an uncertain result in the creation of *p*-maps. When increasing the sample size to above 30 patients (120 simulations), the overall significance was zero for most analyses, but there were still variations in the location of the resulting significance volumes. These variations were in the region between the high- and low-improvement clusters. This is an expected result since voxels in the region between the clusters can be assumed to include a mixture of high- and low-improvement stimulations. The mixture will generate voxel-wise improvement close to the average of 70% in tremor reduction, and thereby not reach significance in the statistical test. Where this average region is located will be a result of the variation in lead localization between samples of patients. The expansion in the superior direction for the high-improvement clusters seen in Figure 5 is a result of more simulations included in each voxel, and thereby more voxels included in the analysis (Figure 3). For analyses with few patients, many of the voxels at the overall outer border will have too few simulations included and they will thereby be excluded.

The significance analysis is impacted by the sample size, but possibly also by the number of permutations. Increasing the number of permutations generates a higher power to the overall *p*-value due to a denser sampling of the null distribution. The statistics are based on the work of Eisenstein et al., which used 200 permutations [7]. However, for statistics on functional imaging, it has been suggested to use at least 1000 permutations [24], and some DBS studies uses this number [4]. As seen in the present study, with few patients the statistics varied more between the samplings even when increasing the number of permutations (Figure 1a–b). Therefore, the variation seen is due to the different sampled individuals in each iteration.

The increasing number of patients in the test will reduce the overall *p*-value and the variation of cluster location and will increase the cluster volume. The difference in volume is partly due to the threshold for excluding voxels with too few samples (Figure 3). With few patients, fewer voxels will be included in the statistics and the overall result will be more uncertain due to less volume covered by the analysis. Interestingly, while the cluster volumes were impacted by the selected thresholding method neither the overall *p*-value nor the DC were. With the 10% threshold, the overall analyzed volume was constant for the region where the threshold increased with sample size (>12 patients) while the cluster volume continued to increase up to about 30 patients. However, even for the region where the total volume was stable, the DC continued to increase generating a more stable location with 60 patients compared to 30 patients (Figure 4 and Figure 5). For the fixed thresholds, the cluster volumes increased for all sample sizes, but with decreasing rate above 25–30 patients, and similar stabilization of location as for the percentage threshold. The selected thresholding method will impact the outer border of the cluster volume. As analyses based on PSMs are often relative, e.g., correlation between the overlap of a patient VTA with the PSM, the selected threshold will have minor effect. Nevertheless, to reduce the effect of outliers and increase the PSM stability, a threshold less than 12 simulations is probably not suitable.

This study concerns monopolar review data, the result of which cannot directly be generalized to other datasets like intra-operative data, or data based on the clinical DBS settings used in the patient’s daily life. For other symptoms, a monopolar review is not as simple since the response to therapy can take much longer times, e.g., up to months for dystonia [25]. Therefore, the clinical DBS settings may be the only data available. One problem with the clinical settings is more pronounced skewness towards high improvement. This skewness can result in a necessity to modify the statistics and the selected improvement value to test against, but also that more samples may be required to achieve similar significance levels. On the other hand, the DBS outcomes for ET patients have relatively low variance and thus for disorders with higher variance in the outcome, a smaller sample may be sufficient. It is important to recognize that the result is a combined effect of the number of patients and simulations. While hundreds of simulations in a single patient generate much information, it cannot be generalized to a patient group. In this study, the number of stimulation settings increased the dataset moderately by including one setting per contact while not causing a large problem of dependency between simulations. However, it can be assumed that PSMs computed from clinical settings could require four times as many patients.

One limitation in this study is the homogeneity of the data. All patients were implanted in the same clinic aiming for the same target, which reduces the variation and results in a bias in spatial analysis. Furthermore, since the dataset is only composed of 61 patients, the dependency between the analysis within a cohort size is higher for larger *n*. This was addressed by using sampling with replacement, and the similar variation over the dataset confirms that this indeed reduces this dependency.

## 5. Conclusions

In conclusion, the resulting *p*-maps in DBS vary to a large extent for small sample sizes both in the size of significance clusters and the overall *p*-value. This variation could not be compensated for by increasing the number of permutations. At sample sizes of around 30 patients (120 simulations), the result stabilizes in significance and volume for this monopolar review dataset. This implies that data analysis based on small samples should be interpreted with caution since small variations in contact position will impact the result.

## Figures and Tables

**Table 1:** Median cluster volumes for 5, 30, and 60 patients and overall variability (mean ± standard deviation) of the IQR over the total range of sample sizes.

	10% Threshold	Fixed Threshold of 5	Fixed Threshold of 12
**High improvement**			
Median volume [mm^3^]			
*n* = 5	54	44	0.3
*n* = 30	277	290	227
*n* = 60	229	490	373
IQR [mm^3^]	96 ± 24	187 ± 64	94 ± 43
**Low improvement**			
Median volume [mm^3^]			
*n* = 5	3.9	4.1	0
*n* = 30	89	154	107
*n* = 60	121	251	177
IQR [mm^3^]	51 ± 14	75 ± 22	50 ± 26

**Table 2:** The number of patients (*n*) where the largest median DC values were reached and the overall variability (mean ± standard deviation) of the IQR over the total range of sample sizes.

	10% Threshold	Fixed Threshold of 5	Fixed Threshold of 12
**High improvement**			
DC max	0.73 (*n* = 57)	0.68 (*n* = 61)	0.70 (*n* = 47)
IQR	0.16 ± 0.06	0.17 ± 0.06	0.15 ± 0.06
**Low improvement**			
DC max	0.69 (*n* = 58)	0.72 (*n* = 61)	0.72 (*n* = 60)
IQR	0.15 ± 0.05	0.15 ± 0.06	0.13 ± 0.06

## Data Availability

The data are not available due to ethical considerations.
